# Differential Methylation of the HPV 16 Upstream Regulatory Region during Epithelial Differentiation and Neoplastic Transformation

**DOI:** 10.1371/journal.pone.0024451

**Published:** 2011-09-07

**Authors:** Svetlana Vinokurova, Magnus von Knebel Doeberitz

**Affiliations:** Department of Applied Tumor Biology, Institute of Pathology, University of Heidelberg, Heidelberg, Germany; Karolinska Institutet, Sweden

## Abstract

High risk human papillomaviruses are squamous epitheliotropic viruses that may cause cervical and other cancers. HPV replication depends on squamous epithelial differentiation. Transformation of HPV-infected cells goes along with substantial alteration of the viral gene expression profile and preferentially occurs at transformation zones usually at the uterine cervix. Methylation of the viral genome may affect regulatory features that control transcription and replication of the viral genome. Therefore, we analyzed the methylation pattern of the HPV16 upstream regulatory region (URR) during squamous epithelial differentiation and neoplastic transformation and analyzed how shifts in the HPV URR methylome may affect viral gene expression and replication. HPV 16 positive biopsy sections encompassing all stages of an HPV infection (latent, permissive and transforming) were micro-dissected and DNA was isolated from cell fractions representing the basal, intermediate, and superficial cell layers, each, as well as from transformed p16^INK4a^-positive cells. We observed fundamental changes in the methylation profile of transcription factor binding sites in the HPV16 upstream regulatory region linked to the squamous epithelial differentiation stage. Squamous epithelial transformation indicated by p16^INK4a^ overexpression was associated with methylation of the distal E2 binding site 1 leading to hyper-activation of the HPV 16 URR. Adjacent normal but HPV 16-infected epithelial areas retained hyper-methylated HPV DNA suggesting that these viral genomes were inactivated. These data suggest that distinct shifts of the HPV 16 methylome are linked to differentiation dependent transcription and replication control and may trigger neoplastic transformation.

## Introduction

Human papillomaviruses are small epitheliotropic DNA viruses that infect squamous cell epithelia of the skin (skin types) and mucosal (mucosa types) surfaces and may cause hyper-proliferative lesions as for example common or genital warts. High risk HPV types (HR-HPVs) are associated with a variety of human cancers particularly of the uterine cervix [1]. Infections with these viruses are extremely widespread among young men and women [2] while related cancers predominantly emerge from few infected basal cells predominantly at the transformation zone of the uterine cervix. These observations strongly suggest that not only the infection of epithelial cells but also mechanisms that govern viral gene expression patterns within the host cells contribute to the control of the papillomavirus life cycle and HR-HPV-related transformation [3]–[5].

The viral genome consists of a double stranded circular DNA molecule that encompasses a set of 6 early genes (E6, E7, E1, E2, E4, E5) that are involved in viral gene expression and replication control, whereas two late genes (L1 and L2) encode the major capsid proteins. During a permissive infection the virus is thought to first infect distinct basal cells [6], where it may reside in a “silenced” or “latent” stage [7]. Some of the initially infected basal cells may enter a permissive life cycle during that offspring viral genomes are replicated in the differentiated squamous cell layers. Finally mature virions are released at the surface of the epithelium. It is not known so far how many of the initially infected cells ever enter the permissive viral life cycle. It may well be that even the majority of infections may end as abortive silenced or latent infections during that no major viral gene expression is ever initiated. Such infections if they occur would not cause any pathology and hence would clinically remain unnoticed.

During a permissive infection in a single infected basal cell the virus appears to express the genes E6 and E7 in a highly controlled pattern that allows for limited local expansion of the respective infected basal cell [8]. Upon infection of the basal cells, HPV genomes are replicated up to 50–100 copies per cell. Following this initial establishment phase, it apparently replicates along with host cell chromosomes, however, without triggering productive viral genome replication as long as these cells remain in the basal or parabasal cell layer. Throughout this maintenance phase in undifferentiated squamous epithelial cells, viral genes are expressed at low levels. Once these cells leave the basal cell layer they differentiate into intermediate squamous epithelial cells. Here, the transcriptional activity of the HPV early genes is substantially increased [8]. This retains the respective cells in a mode capable for DNA replication and triggers replication of the viral genomes [3], [8]. Upon further squamous epithelial differentiation these cells reach the superficial cell layer. Here, the viral early genes are shut off, whereas the two late genes (L1 and L2) become strongly activated (early - late shift) and allow for capsid formation, packaging of the replicated viral genomes and release of mature HPV virions. This complex differentiation dependent viral gene expression and replication mode implies that expression of viral genes is tightly regulated along with squamous epithelial differentiation [9].

Malignant transformation of HR-HPV-infected cells preferentially occurs in cells at the uterine transformation zone. It is usually preceded by characteristic squamous epithelial precursor lesions during that the above outlined viral gene expression pattern is fundamentally changed. The shift from a permissive HPV infection towards cellular transformation is characterized by substantially increased expression of the viral oncogenes E6 and E7 in basal and parabasal cells [10], [11].

Recent work has shown that the overexpression of E7 is accompanied by substantial over-expression of the cyclin-dependent kinase inhibitor p16^INK4a^ that is therefore used as biomarker for HPV-transformed epithelial cells [12]–[14]. The shift from permissive to transforming HR-HPV-infections is regarded as the key event and the rate limiting step in HPV-mediated cellular transformation and carcinogenesis [15]. Several lines of evidence suggested that integration of the HPV genome and disruption of the E2 open reading frame results in loss of the E2 negative regulatory function [16]. However, this concept is in contradiction with findings that demonstrate that many of the fully malignant HPV-associated cancers do not even carry any integrated viral genome copies [17]–[20]. High-grade cervical lesions that encompass integrated viral genomes also carry episomal viral genomes that should be capable to encode the E2 gene product [17]. Integration thus rather seems to be the consequence of chromosomal instability mediated by the deregulated expression of the viral E6 and E7 genes instead of its cause [19], [21].

Thus, if deregulated expression of the E6 and E7 genes and hence the transforming expression mode is considered to be the initiating event of HPV-induced carcinogenesis, it is reasonable to assume that the shift from permissive to transforming infections substantially precedes integration of viral genomes.

Control of gene expression by epigenetic modification of distinct DNA sequences is a fundamental biological process that impacts crucial biological features such as embryonic development, cellular differentiation and aging [22]–[26]. Changes of the epigenetic modification of distinct genes are associated with major human diseases and in particular carcinogenesis [27], [28]. Methylation of HPV genomes have been analyzed in a series of recent publications [29]–[40]. The general observation was that the degree of methylation of the HPV genomes was consistently higher at most sites in carcinomas as compared to dysplastic precancerous lesions. Further data on the influence of DNA methylation in the HPV life cycle came from the studies that focused on methylation of the E2 binding sites (E2BSs) in the HPV 16 URR. [41], [42]. The E2 protein is an important transcriptional regulator of the HPV genome. It mediates its transcriptional control functions through binding to four distinct E2BSs located within the URR. The capacity of the E2 protein to bind E2BSs in vitro is inhibited by methylation of CpG dinucleotides within these E2BSs [43] and therefore inhibits transcriptional activation by the E2 protein [42]. Methylation analysis of E2 binding sites within URR in immortalized HPV-infected epithelial cells (W12 cells) demonstrated that these regions are hypo-methylated upon differentiation in vitro [41], [42]. These observations suggest that the methylation state of the viral genome, and particular that of E2BSs, may vary during the viral life cycle and thus squamous epithelial differentiation. However, the molecular impact of the methylation pattern of HPV genomes during the various stages of the HPV life cycle and also cellular transformation remained largely obscure.

To fill this gap of knowledge we analyzed for the first time the methylation status of the HPV 16 URR in different phases of the viral life cycle linked to distinct phases of the squamous epithelial differentiation pattern in naturally occurring cervical lesions. Specifically, we analyzed the methylation status of CpG dinucleotides in transcription factor binding sites within the HPV 16 URR using DNA preparations isolated from microdissected squamous epithelial cell layers, reflecting basal, intermediate and superficial squamous cell differentation. In addition, we compared the methylation status of the HPV 16 URR in cells that have already undergone the switch to the transformed phenotype as indicated by strong over-expression of p16^INK4a^.

## Results

### Study design

**Figure 1:**
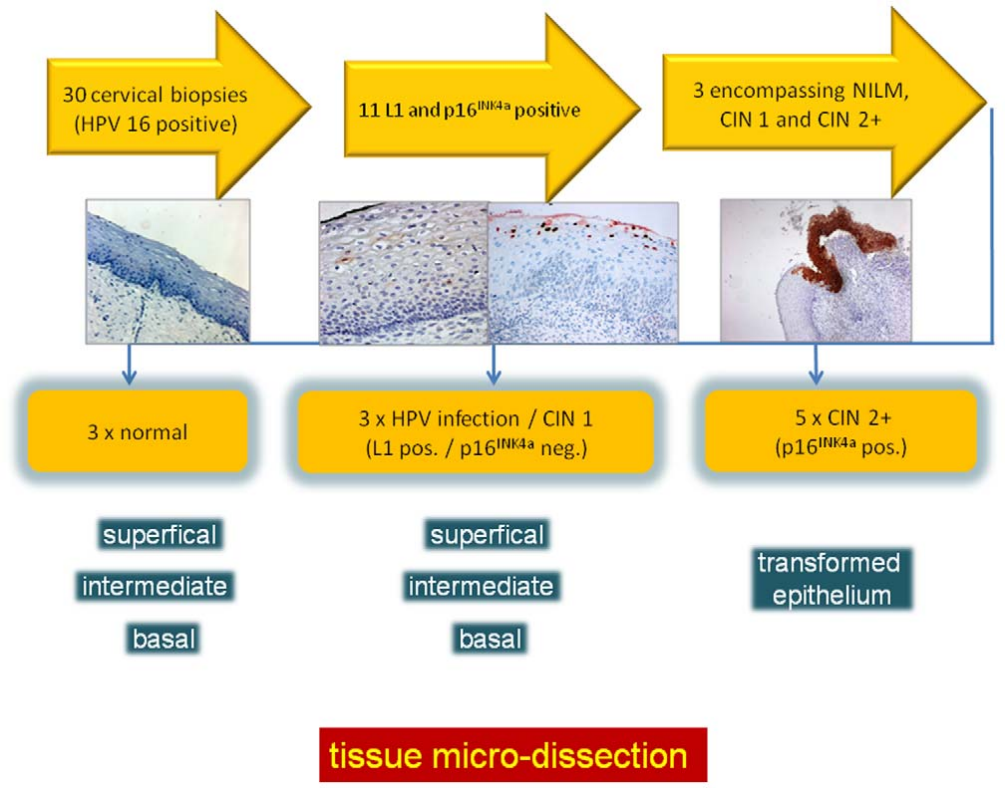
Schematic outline of the recruitment of lesions included into the study. 34 HPV 16 infected cervical lesions were recruited from a gynecopathology archive of cervical punch and cone biopsies. Sections of these lesions were stained with H&E, p16^INK4a^ and L1. 11 of these displayed koilocytes and L1 positive superficial squamous epithelial cells. Of these lesions, three showed a continuum of p16^INK4a^-positive epithelial areas indicating transforming HPV infections. 2 additional biopsies displaying p16^INK4a^-positive epithelial areas were recruited from other biopsies not directly showing this continuum of intra-lesional progression for the in depth methylation analysis as shown in Figure 5.

To test the hypothesis that differential methylation of the viral genome regulates its expression, we analyzed modifications of the methylation status of all CpG dinucleotides in the viral upstream regulatory region (URR) during epithelial differentiation and transformation. Therefore, we selected in a first approach from a large histopathology archive tissue sections of 3 HPV 16 positive samples that encompassed representative parts of all different stages of an HPV-infection (Figure 1, Figures S1, S2, S3, S4, S5, S6, S7, S8, S9, S10). These included: i.) HPV infected epithelium without any morphological changes and no obvious signs of viral replication [44], [45], ii.) cervical intraepithelial low grade lesions (sometimes referred to as flat condylomas) lacking diffuse p16^INK4a^ immuno-reactivity but displaying koilocytes or HPV 16 L1 capsid protein expression in superficial squamous cells as indicator for permissive HPV-infections and iii.) high-grade lesions with diffuse p16^INK4a^ immune reactivity as indicator of transforming HPV 16 infections [12], [13].

**Figure 2:**
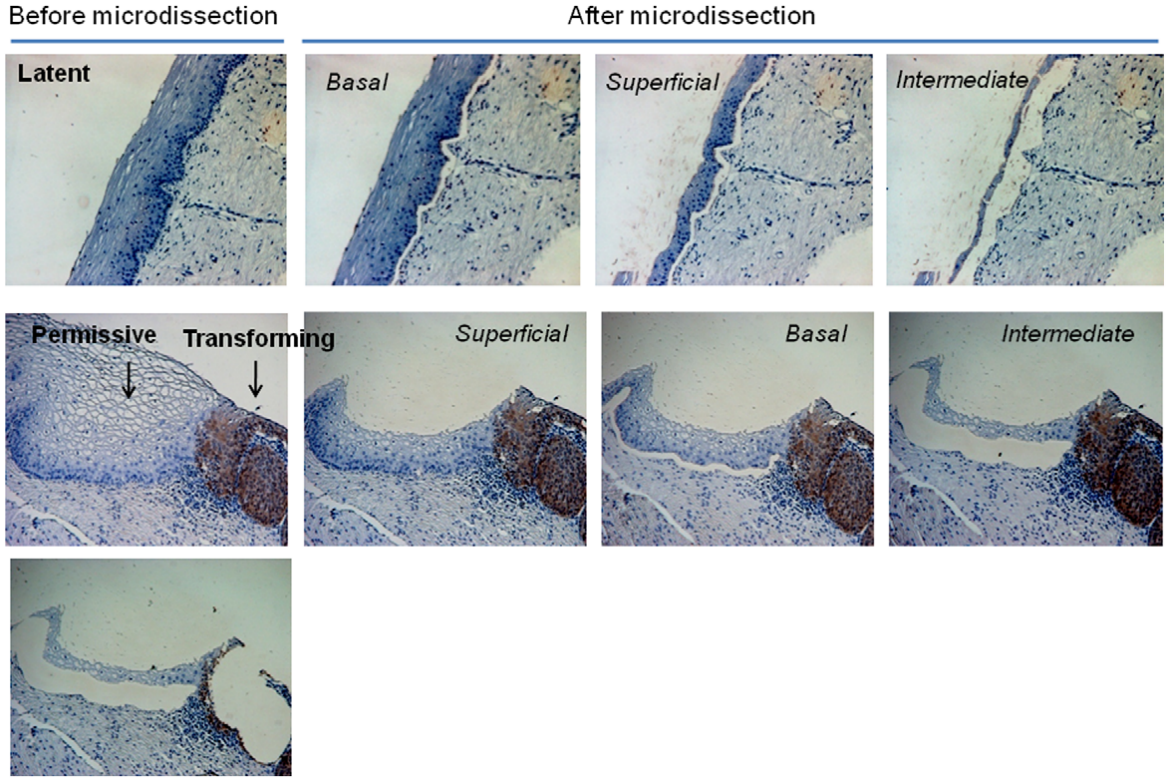
Microdissection of cervical tissues representing latent, permissive, and transforming modes of HPV infection. Laser microdissection of paraffin-embedded section containing areas with: HPV 16 infected epithelium without any morphological changes (normal), permissive infections (i.e. cervical intraepithelial low grade lesions lacking diffuse p16^INK4a^ immuno-reactivity but displaying koilocytes), and adjacent transforming infections (CIN 3 lesions with diffuse p16^INK4a^ immune reactivity). This tissue sections were micro-dissected and DNA was isolated from cell fractions representing the basal, intermediate, and superficial cell layers, as well as from transformed p16^INK4a^-positive cells.

These tissue sections from three independent lesions were micro-dissected and DNA was isolated from cell fractions representing the basal, intermediate, and superficial cell layers (Figure 2, upper and middle panels; Figures S1, S2, S4, S6, S7 and S9), as well as from transformed p16^INK4a^-positive cells (Figure 2, lower panel, right image; Figures S1, S3, S5, S8 and S10). DNA was extracted from each individual microdissected cell sample, treated with sodium bisulfite and amplified with 3 primer pairs covering the HPV16 URR region and analyzed by bisulfite genomic sequencing techniques and the modified Combined Bisulfite Restriction Analysis (COBRA) [46].

### Methylation of the HPV16 URR in latent and permissive infection modes

**Figure 3:**
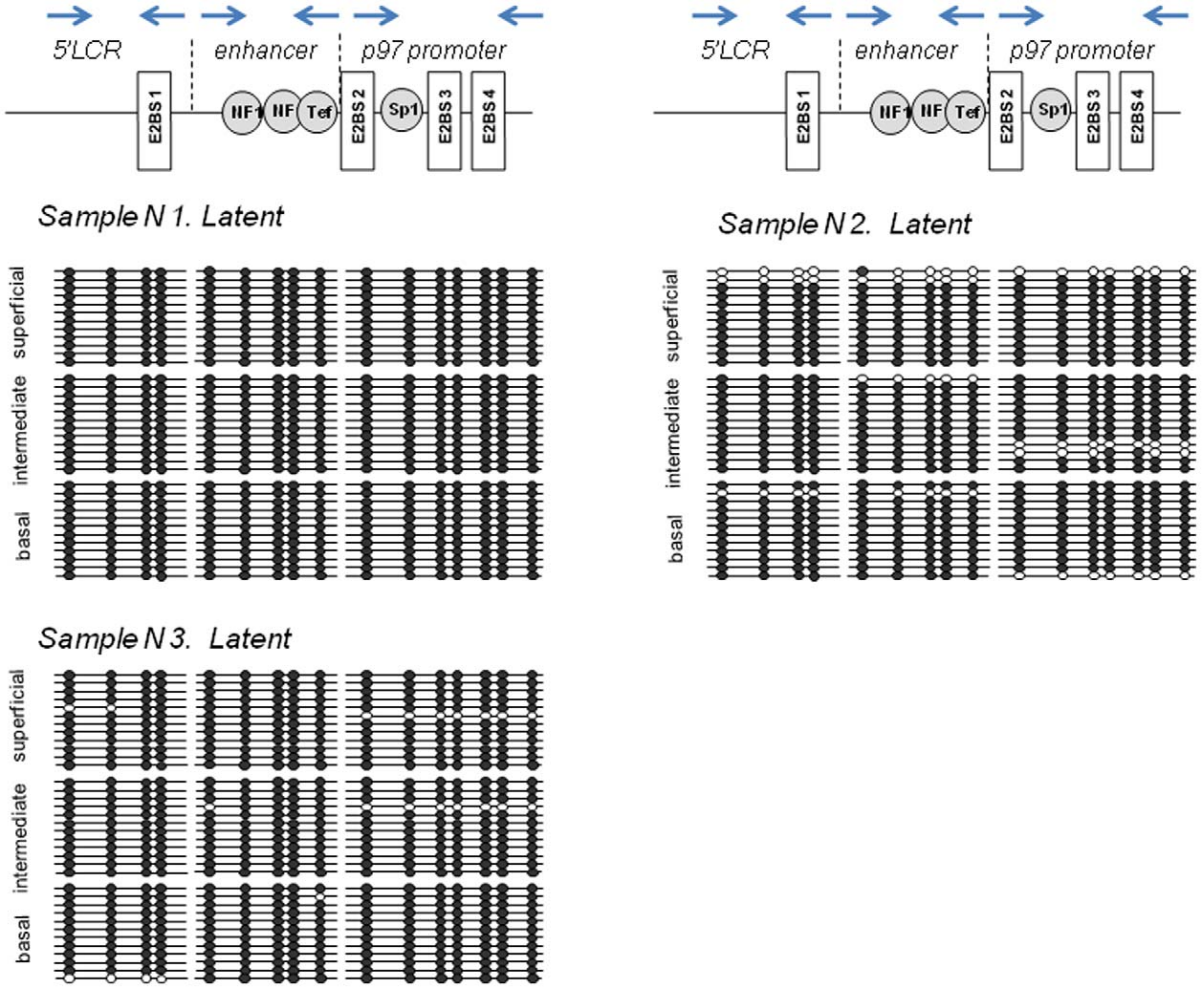
Differentiation dependent HPV16 URR methylation in the latent infections. The methylation status of the HPV16 URR was determined by bisulfite treatment followed by direct DNA sequencing of amplified DNA fragments using 3 sets of primers covering the complete HPV16 URR as indicated by the blue arrows in the upper part of the Figure. DNA derived from laser-microdissected basal, intermediated and superficial layers of the cervical normal HPV 16 infected epithelium was amplified and cloned. 12 individual clones were sequenced to identify the presence and patterns of the methylated CpG dinucleotides. The Figure visualizes schematically the methylation of 16 CpG dinucleotides (positions from 7198 to 161) in the URR of HPV-16 genomes. The analyzed region encompasses 3 functionally distinct segments that have been referred to as the 5′ LCR, the enhancer, and the promoter. Open circles represent an unmethylated CpGs, filled circles represent methylated CpGs.

**Figure 4:**
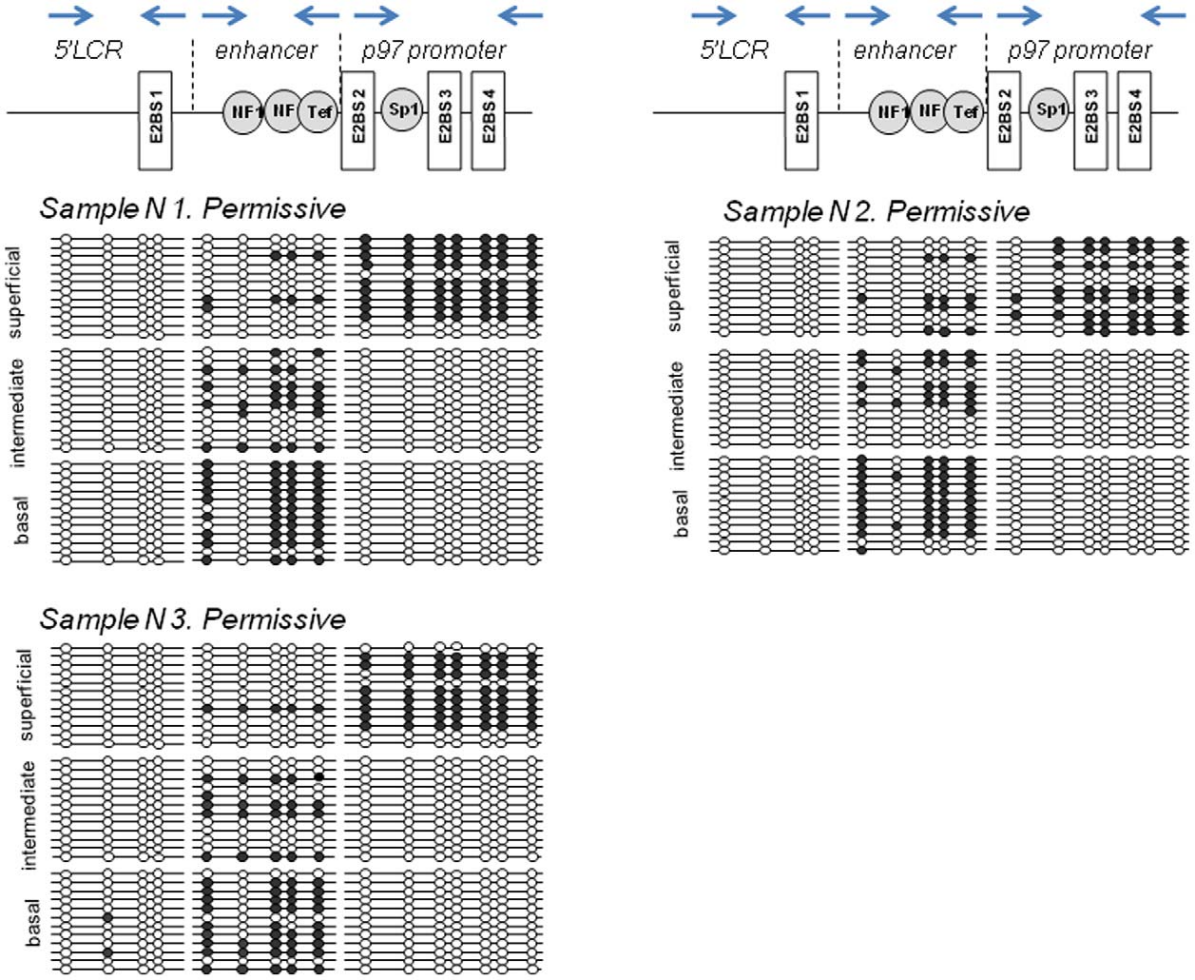
Differentiation dependent HPV16 URR methylation in the permissive infections. The methylation status of the HPV16 URR determined by bisulfite treatment followed by direct DNA sequencing of amplified DNA fragments using 3 sets of primers covering the complete HPV16 URR. DNA derived from laser-microdissected basal, intermediated and superficial layers of the cervical epithelium with permissive HPV16 infection was amplified and cloned. 12 individual clones were sequenced to identify the presence and patterns of the methylated CpG dinucleotides. The Figure visualizes schematically the methylation of 16 CpG dinucleotides (positions from 7198 to 161) in the URR of HPV 16 genomes. Open circles represent unmethylated CpGs, filled circles represent methylated CpGs.

**Figure 5:**
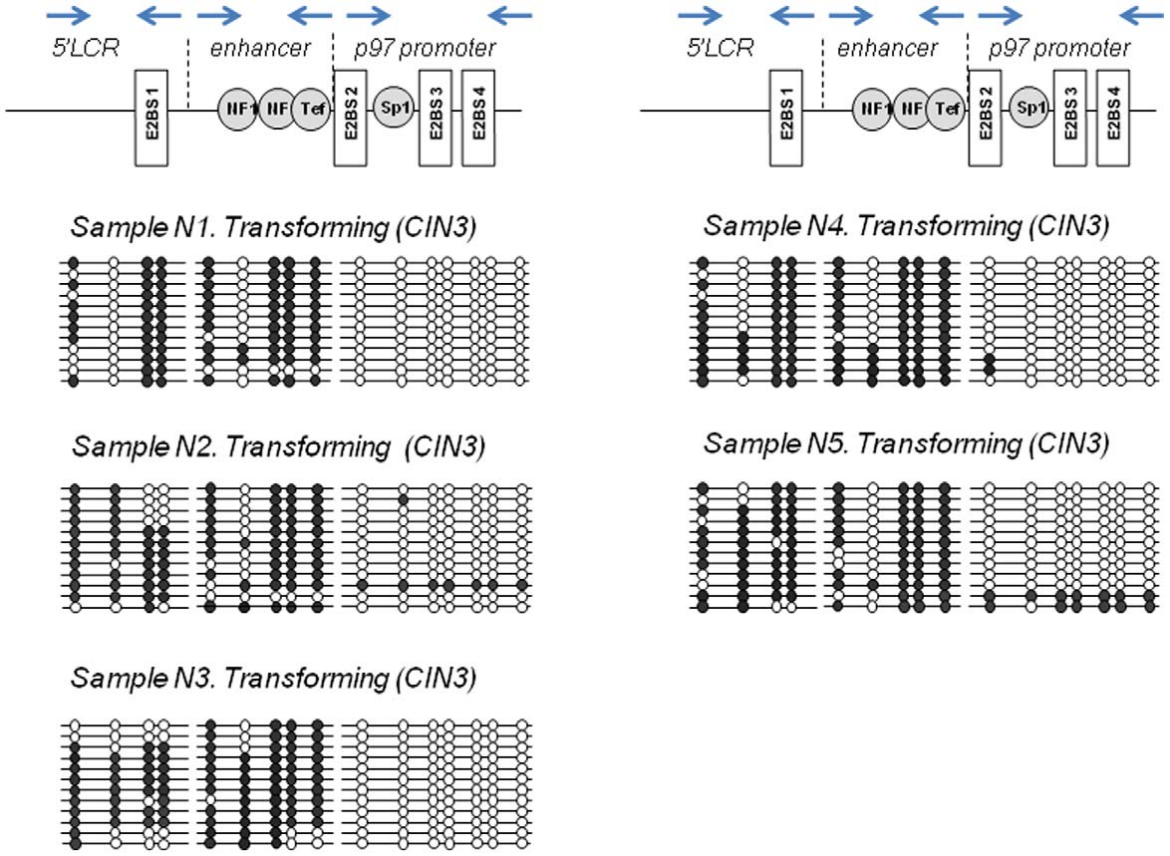
HPV16 URR methylation in the transforming infections. The methylation status of the HPV16 URR determined by bisulfite treatment followed by direct DNA sequencing of amplified DNA fragments using 3 sets of primers covering the complete HPV16 URR. DNA derived from laser-microdissected p16^INK4a^-positive transformed epithelium was amplified and cloned. 12 individual clones were sequenced to identify the presence and patterns of the methylated CpG dinucleotides. The Figure visualizes schematically the methylation of 16 CpG dinucleotides (positions from 7198 to 161) in the URR of HPV 16 genomes. Open circles represent unmethylated CpGs, filled circles represent methylated CpGs.

The analyzed region of HPV16 URR between genomic positions 7198 and 161 contains in total 16 CpG dinucleotides (Figure 3, 4 and 5). It encompasses 3 functionally distinct segments that have been referred to as the 5′ LCR, the enhancer, and the promoter (Figure 3, 4 and 5). The 5′LCR contains 4 CpG dinucleotides including 2 CpGs within the E2BS1. The enhancer contains five CpG dinucleotides including 4 CpG dinucleotides within NFI and TEF-1 binding sites (NFIBS, TEF-1BS). The promoter retains seven CpG dinucleotides, six of which are located within a Sp1 or E2 binding sites (E2BS2, E2BS3, and E2BS4).

**Figure 6:**
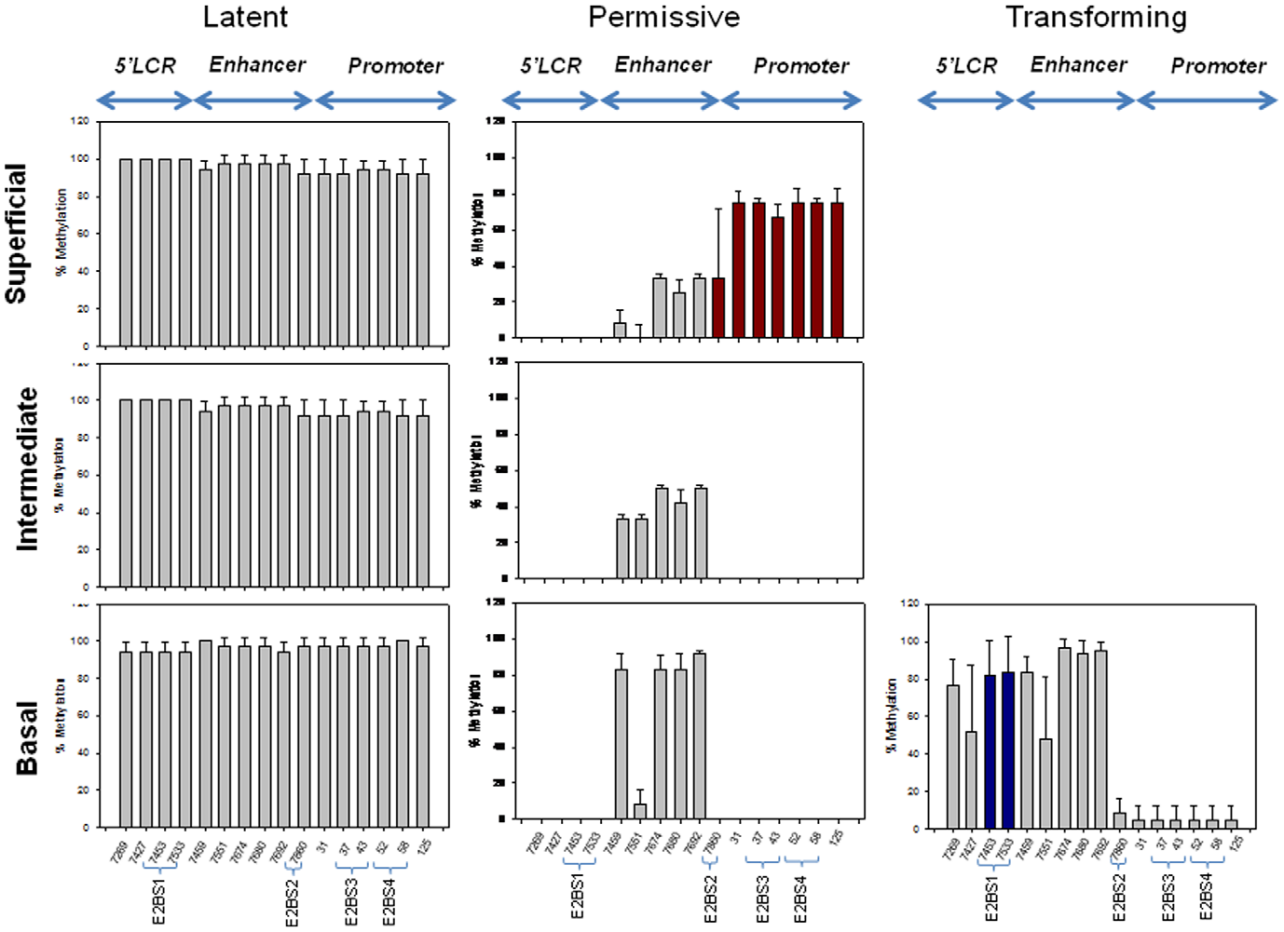
Schematic representation of the relative methylation frequencies of 16 CpG nucleotides within the HPV16 URR. The degree of methylation of 12 independent clones each isolated from basal, intermediate and superficial cell layers from the three latent, permissive and five transforming parts of the cervical epithelium investigated by genomic sequencing as shown in Figure 3, 4 and 5. The y-axis shows the mean percentage of methylated HPV16 genomes at each of the 16 CpG dinucleotides ± standard deviation (SD). Mean values and SDs (three independent lesions per each CpG) were calculated from Sigma Plot 10.0. Nucleotide numbers refer to those of the HPV16 reference clone (GenBank NC_001526).

In squamous epithelial areas that did not display any histopathologic abnormalities (Figure 2, upper panel; Figures S4, S6 and S9) and did not show signs of viral gene expression, all 16 CpG dinucleotides were consistently methylated throughout the whole thickness of the epithelium (Figure 3 and 6). In epithelial areas that display koilocytes and express the L1 capsid protein as histopathologic correlate of permissive HPV infections (Figure 2, middle panel; Figures S1, S2 and S7) [45] the consistent methylation pattern observed in the adjacent normal tissues was substantially changed into a more complex pattern (Figure 4 and 6). The promoter region of DNAs isolated from the basal or intermediate cell layers consistently contained unmethylated CpGs. In the superficial cells, however, most of the CpG dinucleotides of the promoter region were methylated including the E2BSs 2, 3, and 4 as well as the one in the SP1 site. The 4 CpG dinucleotides of the enhancer region including the ones within two NFIBSs and one TEF-1BS were heavily methylated in basal cells but to a lesser extent in the more differentiated intermediate cells (Figure 4 and 6). In the 5′LCR segment all 4 CpG dinucleotides were unmethylated irrespective of the differentiation stage (Figure 4 and 6). Interestingly, the E2BS1 was not methylated in basal, intermediate or superficial squamous epithelial cells during this early stage of the infection.

### Methylation of the HPV16 URR in the transforming infection mode

To determine whether transformation of HPV-infected cells is also linked to changes of the methylation pattern of distinct CpG dinucleotides within the HPV16 URR, we microdissected p16^INK4a^ immunoreactive cells from the same sections (Figure 2, lower panel, right image; Figures S1, S3, S5, S8 and S10) and analyzed the DNA methylation pattern by bisulfite sequencing (Figure 5 and 6). In these cells the 4 CpG dinucleotides within the NFI-BS and the TEF-1BS of the enhancer region were methylated and 7 CpG dinucleotides of the promoter were de-methylated. However, in contrast to the p16^INK4a^-negative areas with permissive infection, 3 CpG dinucleotides in the 5′LCR including the 2 CpG dinucleotides within the E2BS1 were consistently methylated. In addition to these three lesions we further included 2 more lesions displaying diffuse p16^INK4a^ expression and confirmed the latter observation (Figure 5, samples 4 and 5).

To confirm the specific methylation of the E2BS1 in the p16^INK4a^-positive transformed cells detected by bisulfite genomic sequencing, we analyzed the methylation status of 2 CpG dinucleotides within the E2BS1 in additional 34 p16^INK4a^-positive high-grade cervical dysplastic lesions (CIN2 and CIN3) and the corresponding p16^INK4a^-negative control tissues of the same sections using the modified Combined Bisulfite Restriction Analysis (COBRA). Methylation of the CpG dinucleotide within the E2BS1 was detected in HPV genomes of 30 of 34 (88%) p16^INK4a^-positive lesions but only 6 of 34 (17,6%) HPV genomes isolated from p16^INK4a^-negative tissues.

### Effect of E2BS1 methylation on the p97 promoter activity

**Figure 7:**
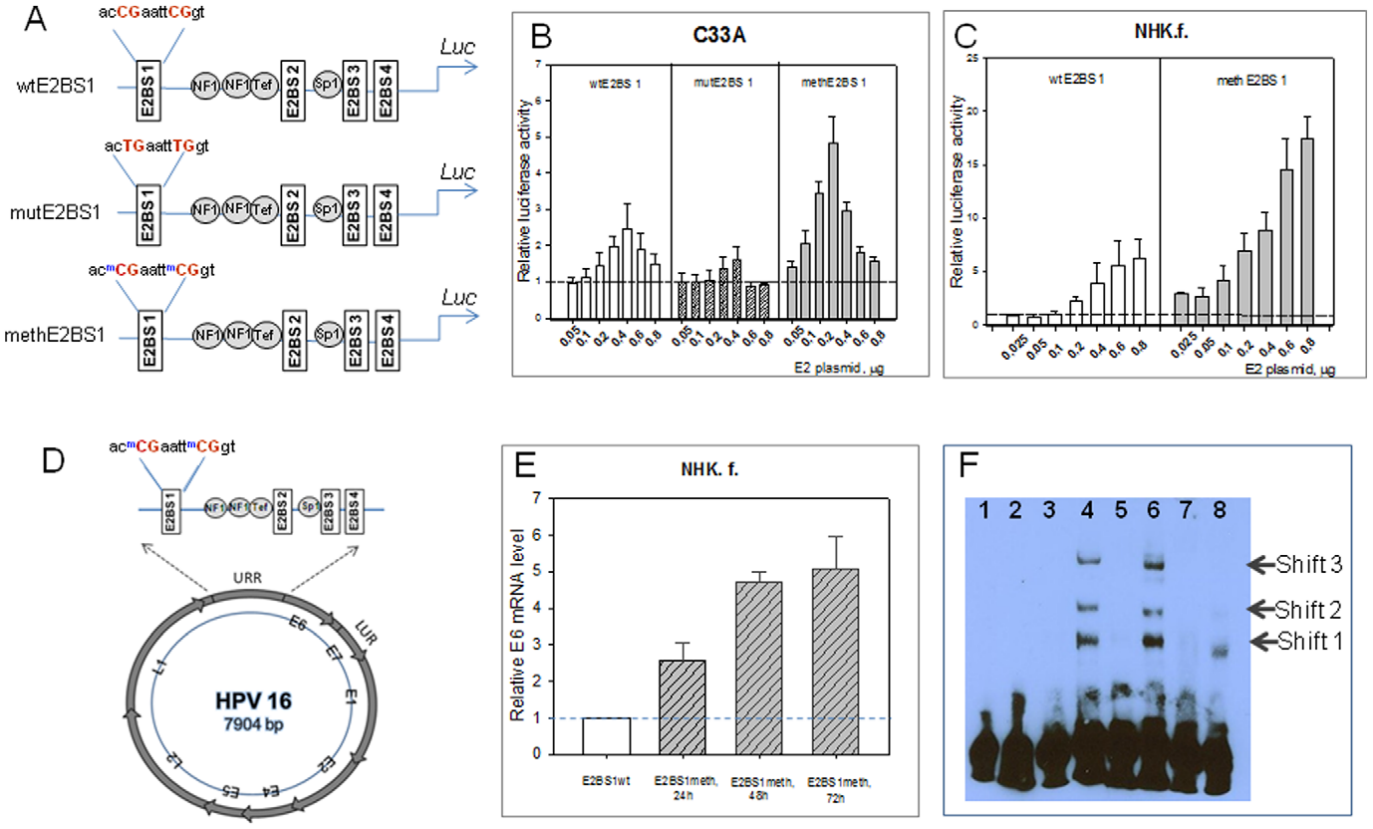
Methylation of the E2BS1 increases the p97 promoter activity. **A.** To measure the impact of methylation of the E2BS1 on the p97 promoter activity site specific modifications within the E2BS1 of LCR HPV16 of pGLuc reporter vector construct were generated using modified oligonucleotides. Three constructs containing either an unmethylated (wt E2BS1), a mutated (mutE2BS1) or a methylated (methE2BS1) E2BS1 were used. **B and C.** These plasmid constructs were co-transfected into C33A and NHK cells and increasing amount of E2 expression vector. The secreted Gaussia luciferase activities were normalized using the corresponding internal ß-galactosidase activities. Each value represents the mean ±standard deviation of at least three independent transfection experiments, each performed in triplicate. **D.** To analyze the impact of methylation of E2BS1 on the early promoter activity in the natural context of the HPV 16 genome where E2 expression is under control of the p97 early promoter, NHK were transiently transfected with full length HPV16 genome (wt or methylated E2BS1). **E.** The effect of selective E2BS1 methylation in full-length HPV16 on E6 gene expression was measured by real-time PCR. Wild type and HPV16 full-length genome and the full length HPV16 genome with 2 selectively methylated CpGs within E2BS1 were transfected into normal human foreskin keratinocytes (NHK.f.). Total RNA from cells 24, 48 and 72 hours after transfection was assayed for the expression of E6 mRNA. Relative luciferase activities were calculated with respect to the values of the wt construct, which was set to 1, for each time point. The data represent the mean of four independent experiments performed with each sample in triplicate with error bars indicating ± S.D. Mean values and SDs were calculated from Sigma Plot 10.0. **F.** To test whether methylation recruits other binding of transcription factors to the region of E2BS1, we performed EMSA analysis using nuclear cell extracts isolated from NHK. Methylated and unmethylated DNA probes spanning the regions containing the E2BS1 were used. The methylated probes showed three shifted bands by adding nuclear cell extracts (line 4, 6). Unmethylated probes were not shifted by the same nuclear extract (lane 3, 5). Competition experiments were performed with non-labeled probes (lines 7 and 8).

The HPV DNA methylation analysis presented above indicated that the CpG dinucleotides within the E2BS1 were almost exclusively methylated in p16^INK4a^-positive high-grade lesions pointing to a functional relevant impact of this specific methylation pattern in HPV-mediated cell transformation. To test whether methylation of these CpG dinucleotides within the E2BS1 is responsible for activation of the early p97 promoter observed during the shift of permissive to transforming HPV infections, we aimed to determine potential functional implications of methylation of these CpG dinucleotides on the activity of the p 97 promoter in transient transfection experiments. The methylated sequences were obtained by PCR as described in material and methods section. In addition, two single-base substitutions in E2BS1 were introduced into the wild-type HPV16 LCR. HPV-negative C33A cells were co-transfected with increasing amounts of an expression vector for HPV16 E2 and a reporter plasmid containing either the entire HPV16 LCR in front of the luciferase gene (wtE2BS1), the LCR with mutations in the E2BS1 (mutE2BS1), or a LCR that was methylated in the E2BS1 (methE2BS1) (Figure 7A). Co-transfection of increasing amounts of the HPV16 E2 expression vector and the reporter construct, wtE2BS1, showed that the p97 promoter was activated by small amounts of HPV16 E2 (Figure 7B). Increasing amounts of HPV16 E2 reduced the promoter under control of the wild-type LCR. Methylation of the E2BS1 significantly induces the luciferase activity in the presence of low amounts of E2, followed by a dose-dependent repression (Figure 7B). The impact of methylation of the E2BS1 was most obvious while using 200 ng of the E2 expression vector. The wild type promoter was 1.9 fold activated, whereas the methylated promoter yielded a 4.8 fold activation. As expected, for the plasmid with C→T mutation in CpG dinucleotides within E2BS1 (mutE2BS1), only slight induction of luciferase activity was observed in the presence of low amounts of E2 expression vector.

To exclude that this effect of the methylation of the E2BS1 on the p97 promoter activity depends on the cell type (C33A cells) that was used we repeated these experiments using normal human foreskin keratinocytes for transient transfection experiments. Here, again we observed strong activation of the p97 promoter while using the construct with the methylated E2BS1 (Figure 7C).

Next we aimed to test these regulatory features under experimental conditions in that E2 expression was driven by the same genome controlled by the p97 early promoter as it is seen in the natural situation. We therefore used quantitative real time PCR to measure the amount of E6 transcripts transcribed from full-length HPV 16 genome with either unmethylated wild type (wtE2BS1) or methylated (methE2BS1) CpG dinucleotides in the E2BS1 upon transfection of the respective plasmids into human primary foreskin keratinocytes (Figure 7D). The amount of E6 transcripts was determined from total RNA preparations extracted from cells 24, 48 and 72 hours after transfection. The results show a 2.6, 4.8 and 5.2-fold increase in E6 transcript levels for genomes carrying the methE2BS1 compared to genomes with wtE2BS1 genomes after 24, 48 and 72 hours, respectively (Figure 7E). These experiments thus confirmed the results obtained in the co-transfection experiments (Figure 7B and 7C) indicating that methylation of the CpG dinucleotides within the E2BS1 result in substantial activation of the promoter activity of the p97 promoter.

### Binding of alternate transcription factors to the methylated E2BS1

An earlier report by Thain et al. suggested that E2 does not bind to methylated E2BSs [43]. However our data revealed that the promoter activity of constructs encompassing methylated CpG dinucleotides in the E2BS1 was substantially enhanced if compared to the unmethylated form. This effect was depending on co-expressed E2 (Figure 7B and 7C). We therefore hypothesized that additional cellular factors may be involved in the E2-mediated regulation of the p97 promoter activity via either the methylated or unmethylated E2BS1.

We used EMSA analyses with nuclear cell extracts isolated from different HPV-negative squamous epithelial cell lines (normal foreskin keratinocytes, C33A, HT3) to test whether differential methylation of the two CpG dinucleotides within the (E2BS1) results in binding of alternate transcription factors to the methylated in comparison to the unmethylated E2BS1. Methylated and unmethylated DNA probes spanning the regions at nt 7444–7468 were 3′ end-labeled with biotin. The methylated probes showed three shifted bands (Figure 7F, lines 4, 6). In contrast, unmethylated probes were not shifted by any of the nuclear extracts (Figure 7F, lanes 3, 5). Competition experiments were performed with full-length, non-labeled probes as illustrated in Figure 7F (lines 7, 8). Binding to the methylated probe was competed by pre-incubation with a 100-fold excess of a methylated oligonucleotide (Figure 7F, lane 8), suggesting that a yet uncharacterized protein complex binds specifically to this methylated sequence.

The EMSA experiments indicate that a complex of not yet in detail characterized proteins apparently bind to the region of methylated E2BS1, but fails to bind to the unmethylated E2BS1. This experiment supports the hypothesis that binding of cellular factor(s) might be substantially influenced by the methylation status of respective CpG dinucleotides. Therefore, methylation of the E2BS1 (Figure 5) of the HPV 16 URR observed in the transforming mode of HPV-infection (p16^INK4a^-positive lesions) may have a direct influence on the transcriptional activity of the HPV 16 URR by binding of a yet uncharacterized complex of transcription factors.

## Discussion

Previous reports suggested that the HPV genome is differentially methylated during progression from simple infected to transformed cells [29], [30], [33]–[35], [37], [38], [40], [47], suggesting that differential methylation of the viral genome may somehow be involved in the regulation of viral gene expression and possibly also replication control. Alterations of the HPV-methylome were observed particularly in the URR and L2 and L1 gene in high grade pre-cancer and invasive cancer suggesting that the lack of expression of these genes may be attributed at least in part to increasing methylation of the respective parts of the viral genomes [29], [34], [35], [37], [40], [47]–[49]. The E2BS2 to 4 were also found to be increasingly methylated in more advanced dysplasia or invasive carcinomas [34]–[37], [40]. Although it has not been analyzed in detail, the increased methylation pattern in some of those studies might well be explained by integration of the viral genomes into the host cell chromosomes. Genomic integration of viral genomes has been repeatedly shown to be associated with hyper-methylation of the viral genomes in the integrated context [35], [38], [50], [51].

The permissive life cycle of HPV is restricted to preneoplastic lesions and essentially coupled to squamous epithelial differentiation. In this report we for the first time used DNA isolated from microdissected squamous epithelial cells reflecting various differentiation conditions of HPV-infected squamous epithelial cells of the uterine cervix. These included a.) normal squamous epithelium adjacent to lesions induced by HPV 16 infections, b) areas of active viral replication reflected by koilocytes or the production of mature viral particles as indicated by HPV L1 expression in the superficial squamous epithelial cell layers, and c.) areas of the neoplastic transformation of the squamous epithelial host cells indicated by the strong over-expression of the cyclin dependent kinase inhibitor p16^INK4a^ [12], [13].

The data described here reveal three major novel findings. 1.) There are epithelial cells adjacent to HPV 16 induced cervical lesions that do not show any evidence of an ongoing HPV infection (Figure 2, Figures S4, S6, and S9) but carry viral genomes methylated in all 16 CpG dinucleotides of the HPV 16 URR analyzed (Figure 3 and 6). This finding strongly suggests that the extensive methylation of the HPV 16 genome in these cells prevents viral gene expression and replication, rendering the viral genomes inactive or “silent” passengers in these cells without causing any cytopathic effects.

2.) In lesions characterized by koilocytes as hallmark for permissive HPV infections or that stain positive for the L1 capsid protein (Figure 1, Figures S1, S2 and S7) there are substantial differences of the HPV methylome depending of the degree of differentiation of the squamous epithelium. In the basal and parabasal cells there is methylation of cellular transcription factor binding sites within the viral enhancer element, whereas all E2BS in the promoter and the 5′upstream regulatory region are un-methylated. With maturating differentiation the degree of methylation of the transcription factor binding sites in DNA isolated from the intermediate cell layers within the enhancer region gradually decreased (Figure 4 and 6). In sharp contrast to these findings, all CpGs in the viral promoter region were completely methylated in DNA samples isolated from microdissected superficial cells that displayed the fully differentiated squamous epithelial phenotype and did express the late viral gene product L1, whereas most other CpG dinucleotides remained unmethylated even in the very superficial cells.

3.) The third major observation was that HPV-transformed p16^INK4a^-positive basal and parabasal squamous epithelial cells (Figure 2, Figures S1, S3, S5, S8 and S10) consistently displayed methylation of the two CpG dinucleotides within the E2BS1 (Figure 5 and 6). Taken together these data suggest that there are distinct changes of the HPV methylome in relation to squamous epithelial differentiation. Moreover the progression from non-transforming infection modes (p16^INK4a^-negative) to the transforming infection mode (p16^INK4a^ overexpression in at least basal and parabasal cells) was associated with the consistent methylation of two defined CpG dinucleotides within the E2BS1.

This was surprising, since the E2BS1 is known to activate the HPV URR [52]–[54]. Methylation of this site and reduced binding of E2 to this site was thus expected to suppress the activity of the HPV 16 URR. This in turn should have resulted in decreased but not increased expression of the downstream early genes E6 and E7 as it is consistently observed in the transforming (p16^INK4a^-positive) HPV 16 transcription mode. Transient transfection experiments reported here with unmethylated and selectively methylated E2BS1 revealed, however, that E2 dependent activation of the methylated reporter construct was substantially higher if compared to the unmethylated construct (Figure 7B, 7C and 7E). Methylation of E2BS1 thus appears to significantly increase the p97 promoter activity. This indeed shows that methylation of the E2BS1 may result in strong activation instead of inhibition of the HPV 16 URR. This is in line with the consistent observation that the shift towards the transformation is characterized by enhanced expression of the E6 and E7 genes in basal and parabasal squamous epithelial cells. Interestingly, a recent report also suggested that CpG methylation in another context may create binding sites with altered binding features and may lead to substantial activation of the respective promoter elements [55].

To test whether the observed methylation of the 2 CpG dinucleotides within the E2BS1 may indeed affect the binding properties of putative cellular transcription factors we performed EMSA assays and observed that the fragment encompassing the methylated E2BS1 attracted a protein complex that may be involved in the substantially altered transcriptional regulatory features of this part of the HPV 16 URR found in the transforming mode of HPV infections. This complex is currently being investigated in detail in ongoing experimental work.

Taken together, the data presented here demonstrate that shifts of the HPV methylome are linked to the various stages of squamous epithelial differentiation. Moreover, the transition towards the transforming mode of HPV infections appear to be linked to distinct shifts of the methylation pattern in the HPV URR that are apparently activating it and may provide a molecular explanation for the substantially enhanced expression of the viral E6 and E7 oncogenes in this advanced phase of persistent HPV infections.

## Materials and Methods

### Origin of samples

From the archive of a large clinical pathology laboratory we collected paraffin blocks of sections that displayed all stages of an HPV infection, i.e. CIN 1, CIN 2 and CIN 3 lesions as well as adjacent histologically normal mucosa. DNA was isolated using QIAamp DNA FFPE Tissue Kit (Qiagen). HPV detection and genotyping was performed using the Multiplex HPV genotyping Kit according to the manufacturer's instructions (Multimetrix GmbH, Regensburg). After PCR amplification, the fluorescence-labeled bead-bound probes were measured in a Luminex LX 100 analyzer. From these samples only the HPV 16 positive were selected and prepared for microdissection. Recruitment of the samples was part of an on-going study to identify molecular parameters of persistent HPV infections that was approved by the ethics committee of the Landesärztekammer Baden-Württemberg (136/95). All samples used in this study were retrieved from a routine pathology archive in a completely anonymous way. There was no possibility for the laboratory performing the research investigation to track back the identity of the respective patients. They were older than three years and supposed to be discarded as part of the routine diagnostic process. It was therefore not possible to obtain written informed consent of the patients.

### Immunohistochemistry and Microdissection

Polyethylene membranes (SL-Microtest, Jena, Germany) were cut to size and mounted on non-charged Superfrost Color glass slides (Menzel-Glaser, Braunschweig, Germany). Slides for immunohistochemistry staining were treated with 8% 3-aminopropyltriethoxysilane (APES) solution in acetone. Serial 5-µm paraffin sections were cut with a fresh knife, mounted on prepared slides, and incubated at 60°C for 2 hours to achieve better tissue adhesion to the membrane. Deparaffinization was carried out by incubation in xylene (7 minutes), followed by washing and rehydration in ethanol (99.9% ethanol 5 minutes, 96% ethanol 5 minutes, 70% ethanol 5 minutes). Immunhistochemical staining for the expression of p16^INK4a^ was performed using the CINtec p16^INK4a^ Histology kit for manual staining (mtm laboratories, Heidelberg, Germany) according to manufacturer's instructions. Cytospin preparations of the HPV18 positive cervical cancer cell line HeLa were used as positive controls.

Immunostaining for L1 was perfomed using the Cytoactive kit for HPV L1 (Cytoimmun Diagnostic GbmH, Pirmanses, Germany) as prescribed by the manufacturer. Counterstaining was performed with Mayer's Hematoxylin (DakoCytomation).

Selected p16^INK4a^-positive clusters, as well as p16^INK4a^-negative non-tumour tissue (basal, parabasal, and superficial) cells, were collected separately in the cap of a microfuge tube by laser pressure catapulting using the PALM® Robot-Micro Beam for microdissection.

### DNA preparations and mapping of methylated CpGs in the URR of HPV16

DNA was isolated using QIAamp DNA FFPE Tissue Kit (Qiagen). The methylation status of the HPV16 URR was determined by bisulfite treatment. Genomic DNA from microdissected specimen or cell line was bisulfite-modified using the EZ DNA methylation kit (Zymo Research, Orange, CA) according to the manufacturer's instructions. One microgram of DNA from the Caski and SiHa cell lines was used as control and treated concurrently with the samples to ensure complete bisulfite treatment. After treatment, the resulting bisulfite-modified DNA was eluted in 30 µl of the kit elution buffer and stored at −20°C. Five microliter of the bisulfite-modified DNA was used for each PCR reaction. A nested PCR system was developed using primers that span the URR of HPV 16 (from nt 7198 to nt 161; NC_001526) (Table 1.).

**Table 1:** Primers used for bisulfite genomic sequencing of the HPV16 URR.

	Primer	Sequence 5′ to 3′
**5′ LCR**	5′ LCR16 _for	TTTGTATGTGTTTGTATGTGT
	5′ LCR16 _rev1	TTAAACCATAATTACTAACATAA
	5′ LCR 16_rev2	ACATTTTATACCAAAAAACATA
**Enhancer**	Enh16_for	TAGTTTTATGTTAGTAATTATGGTT
	Enh16_rev1	ATTAACCTTAAAAATTTAAACC
	Enh16_rev2	AAAAATTTAAACCTTATACCAA
**P97 early promoter**	p97 Prom16_for1	TTGTATGTGTTTGTATGTGT
	P97 Prom16_for2	GGTTTAAATTTTTAAGGTTAAT
	P97 Prom16_rev	ACAACTCTATACATAACTATAATA

PCR reaction mixtures were performed in a total of 50 µl containing 10× PCR buffer, 1.5 µl 50 mM MgCl_2_, 1 µl 10 mM deoxynucleotide triphosphates, 0.5 µl of each PCR primer (25 µM) , 2.0 U Platinum Taq (Invitrogen) and 5 µl of the bisulfite modified DNA. Amplification conditions were as follows: initial denaturation at 94°C for 2 min followed by 40 cycles and 30 cycles for the nested PCR of 94°C for 40 s, annealing at 50°C for 30 s, extension at 72°C for 1 min and finally 72°C for 4 min. PCR products were electrophoresed and isolated from 1.2% agarose gels stained with ethidium bromide. Isolated PCR products were then purified by QIAquick Gel Extraction Kit (Qiagen, Hilden) according to the manufacturer's instructions. Purified PCR fragments were cloned the TA Cloning System (Invitrogen) and 12 individual clones were sequenced to identify the presence of methylated CpGs within the HPV 16 LCR. Sequencing of bisulfite modified sample DNA was performed using the BigDye terminator sequencing kit (Applied Biosystems, Foster City, CA) according to the manufacturer's recommendations. The sequencing PCR products were analyzed on the ABI Prism 3100 Genetic Analyzer. The degree of methylation of the 12 independent clones each isolated from basal, intermediate and superficial cell layers from the three latent, three permissive and 5 transforming epithelial regions was analyzed by using the Systat statistical data evaluation software (SigmaPlot version 10.0).

### Analysis of the methylation status of the HPV16 E2BS1 using the Combined Bisulfite Restriction Analysis (COBRA)

In order to determine the methylation status of the E2BS1 [7450-acCGaattCGgt-7461], distal to p97 promoter of HPV16 we used modified Combined Bisulfite Restriction Analysis (COBRA) [46]. DNA isolated from p16^INK4a^-positive and p16^INK4a^-negative regions was bisulfite treated using the EZ DNA methylation kit (Zymo Research, Orange, CA) according to the manufacturer's recommendations. After treatment, 5 µl of aliquot were amplified in 50 µl of solution containing 1× buffer, 1.25 mM deoxynucleotide triphosphate mixtures, 2.5 pmol of each primer, and 1.5 unit of Taq DNA polymerase (Life Technologies, Inc.). PCR was carried out as follows. After a hot start, the cycling parameters were: 94°C for 40 s, 50°C for 30 s, and 72°C for 60 s for 45 cycles and final elongation at 72°C for 4 min. Primers used for COBRA were as follows: HPV16-E2BS1for 5′-AATTGTGTTGTGGTTATTTATTG-3′ and HPV16-E2BS1rev 5-CAAATTTAAACCATAATTACTAAC-3′. After amplification, PCR products were digested with the restriction enzyme EcoRI (New England Biolabs). The EcoR I recognizes E2BS1 sequences unique to the methylated and bisulfite-converted DNA. DNA was electrophoresed in 2% agarose gels. The gels were stained with ethidium bromide.

### Generation of constructs

The LCR (nt 7008–124) of the HPV16 reference clone (sequence identical to that published in the GenBank under the accession number NC_001526) was PCR-amplified with Pfu polymerase (Stratagene) and specific primers containing restriction enzyme recognition sequences for HindIII (5′-ATCAAGCTTGACCTAGATCAGTTTCCTTTAGGAC-3′), and BamHI (5′-ATCGGATCCTCCTGTGGGTCCTGAAACATTGCAG-3′). Restriction-digested PCR products were cloned into pGLuc-Basic Vector (New England Biolabs) giving pGLuc-LCR16 reporter vector construct. The constructs were verified by DNA sequencing. Site specific modifications within the LCR HPV16 of pGLuc-LCR16 reporter vector construct at the 7450-acCGaattCGgt -7461 site was generated using modified oligonucleotides purchased from Thermo Scientific. Modified oligonucleotides contained either a mutated (5′-GCTTCAAC**T**GAATT**T**GGTTGCATfGC-3′), methylated (5′-GCTTCAAC**^M^**CGAATT**^M^**CGGTTGCATGC-3′) or an unmethylated (5′-GCTTCAACCGAATTCGGTTGCATGC-3′) CpG dinucleotides at the site of interest and spanned 25 bps on either side of the site. PCR was carried out according to the protocol in the QuickChange site-directed mutagenesis kit (Stratagene) using 1 ng/reaction of the LCR HPV16 luciferase reporter as template. Following PCR the reaction was treated with DpnI for one hour to ensure that the plasmid used for template was digested. Products were purified using the PCR purification kit (Qiagen). Concentrations of the product were determined by ethidum bromide gel electrophoresis quantification. These products were then used directly for transfection using Fugene HD method (Roche Applied Science).

In vitro DNA methylation was accomplished with CpGmethylase (SssI methyltransferase), by following the procedure recommended by New England Biolabs, the commercial provider of SssI. Completion of DNA methylation was assessed by digestion with the Hpa II restriction enzyme, which cleaves at its recognition sequence only if the DNA is not methylated at the cytosine residue within it.

For the generation of methylated and unmethylated LCR HPV16 containing DNA fragments, the double-stranded LCR 16 DNA fragment, which was or was not subjected to in vitro methylation with the *Sss*I CpGmethylase, was cloned into the HindIII and BamHI-linearized reporter plasmid pGluc-promoter (New England Biolabs). Ligated products were purified using the PCR purification kit (Qiagen). Two micrograms of the ligated products generated with methylated or unmethylated LCR was transfected into C33A cells. Mammalian expression vector pFLAG-CMV-3 (Sigma) was used to express FLAG-tagged E2 HPV16 protein. The plasmid pFLAG-CMV-3-E2HPV16 was constructed by cloning the HPV-16 E2 open reading frame, generated by PCR amplification using low-grade HPV16 positive cervical lesions as template with a HindIII site-containing sense primer (5′-*ATCAAGCTT*ATGGAGACTCTTTGCCAACG-3′) and a BamHI site-containing antisense primer (5′-*ATCGGATCC*TGTCATATAGACATAAATCCAG-3′) into pFLAG-CMV-3. FLAG–E2 expression was confirmed by Western blotting with anti-FLAG M2 antibodies (Sigma) (Figure S11).

### Cell culture, transfections and luciferase assay

Normal human keratinocytes (Promocell) were grown in K-SFM keratinocytes defined serum-free medium (Invitrogen).http://www.pubmedcentral.nih.gov/articlerender.fcgi?artid=1462964 - R10 The human cervical cancer derived cell line C33A was grown in Dulbecco's modified Eagle medium supplemented with 10% fetal bovine serum. Cells were transfected by a method using Fugene HD (Roche) liposomes as specified by the manufacturer. Secreted Gaussia luciferase activity was determined using the Gaussia Luciferase Assay Kit (New England Biolabs), according to the manufacturer's instructions. To avoid harvesting luciferase activity from detached cells, supernatants were spun at 14,000 rpm for 5 minutes. 10–20 µl of supernatant from a 48-well plate (total volume 0.5 ml) was added to 100 µl of GLuc Substrate prior to analysis in a luminometer (Tecan 3010). The pSV-ß-Galactosidase Control Vector (Promega) was used as ß-galactosidase internal controls for transfection efficiency, and all luciferase activity measurements were corrected for ß-galactosidase activity.

### Real time RT-PCR

Total RNA was isolated with RNeasy kit (Qiagen). 1 µg of total RNA was used for synthesis of first strand cDNA with random primer and the Superscript III system (Invitrogen). Each 20 µl real time Q-PCR reaction mixture contained 1× SYBR® Green PCR Master Mix (Applied Biosystems, Foster City, CA), 2.5 µl of cDNA and 0.3 µM of each primer. E6 gene expression was quantified using with E6 forward (5′-AAGCAACAGTTACTGCGACGTGAG-3′) and E6 reverse (5′-CGGTCCACCGACCCTTATATT-3′) primers. The beta-actin gene was used as a reference. The amplification was carried out according to the conditions suggested by the manufacturer (initial denaturation at 95°C for 10 min and 40 cycles of 95°C for 15 s and 60°C for 1 min) using an ABI Prism 7700 Sequence Detection System (Applied Biosystems, Foster City, CA). Each measurement was performed in triplicate and the threshold cycle numbers (Ct) were measured. The copy number was generated from the Ct value.

### Electromobility shift assay (EMSA)

For EMSA, complementary 25-mer methylated oligonucleotides, including E2BS 1 (most distal to the P97 promoter of HPV-16; E2BS1 sense, 5′-GCTTCAAC**^M^**CGAATT**^M^**CGGTTGCATGC-3′, and E2BS1 antisense, 5′-GCATGCAAC**^M^**CGAATT**^M^**CGGTTGAAGC-3′) (**^M^**C = 5-methylcytosine), were end-labeled with biotin using a biotin 3′-end DNA-labeling kit (Pierce) and annealed. The unmethylated probes were assembled with two complementary oligonucleotides of identical sequence except that cytosine replaced 5-methylcytosine on both strands. Binding reactions were carried out by using a Light Shift chemiluminescent electrophoretic mobility shift assay (EMSA) kit (Pierce) according to the manufacturer's instructions. Twenty-microliter reaction mixtures containing 10 mM Tris, 50 mM KCl, 1 mM dithiothreitol, 50 ng of poly(dI-dC)/µl and 5–10 µg of nuclear extract , and 20 fmol labeled, double-stranded oligonucleotide were incubated for 30 min at room temperature. Reaction mixtures lacking protein or containing cold competitor (4 pmol unlabeled oligonucleotide) were included as controls. Reaction products were resolved on 6% Tris-borate-EDTA-polyacrylamide gels at 90 V in 0.5% Tris-borate-EDTA buffer: 100 mMTrisHCl, 100 mM borate, and 2 mM EDTA, pH 8.3. Proteins were then transferred to Hybond N+ (Amersham Pharmacia Biotech), and biotinylated DNA was detected by chemiluminescence per the manufacturer's instructions.

## Supporting Information

Figure S1 **Sample N1.** Regions with HPV16 permissive and transforming infections. Before and after microdissection (left upper and lower images). The p16^INKa^-negative region displaying koilocytes (upper,right image) and L1 expression (lower right image) as indicator for permissive infection. This area corresponds in pathology terms to a flat condyloma.
(TIF)

Figure S2 **Sample N2.** Region with HPV16 permissive infection. Before and after microdissection (left upper and lower images). The p16^INKa^ -negative region displaying koilocytes (upper right image) and L1 expression (lower right image) as indicator for permissive infection. The p16^INK4a^-negative parts here again correspond to a flat condyloma.
(TIF)

Figure S3 **Sample N2.** Region with HPV16 transforming infection. Before and after microdissection (left upper and lower images). The p16^INKa^ positive region (right image) of transformed epithelium.
(TIF)

Figure S4 **Sample N1.** Region with HPV16 latent infection. Before and after microdissection. The p16^INKa^-negative region without morphological changes of the epithelium and no signs of the viral replication (no koilocytes and no L1 expression).
(TIF)

Figure S5 **Sample N3.** Region with HPV16 transforming infection. Before and after microdissection. The p16^INKa^-positive region (right image) of transformed epithelium.
(TIF)

Figure S6 **Sample N2.** Region with HPV16 latent infection. Before and after microdissection. The p16^INKa^-negative region without morphological changes of the epithelium and no signs of the viral replication (no koilocytes and no L1 expression).
(TIF)

Figure S7 **Sample N3.** Region with HPV16 permissive infection. Before and after microdissection (left upper and lower images). The p16^INKa^-negative region displaying koilocytes (upper right image) and L1 expression (lower right image) as indicator for permissive infection, corresponding again to a flat condyloma.
(TIF)

Figure S8 **Sample N4.** Region with HPV16 transforming infection. Before and after microdissection. The p16^INKa^ -positive region (right image) of transformed epithelium.
(TIF)

Figure S9 **Sample N3.** Region with HPV16 latent infection. Before and after microdissection. The p16^INKa^-negative region without morphological changes of the epithelium and no signs of the viral replication (no koilocytes and no L1 expression).
(TIF)

Figure S10 **Sample N5.** Region with HPV16 transforming infection. Before and after microdissection. The p16^INKa^-positive region (right image) of transformed epithelium.
(TIF)

Figure S11 **Expression of HPV-16 E2 protein in C33A cells.** The HPV 16 E2 protein was expressed from the pFLAG-CMV-3 vector. After 48 h of incubation, the transfected cells were disrupted in 200 µl of lysis buffer. Forty microliters of each lysate was subjected to sodium dodecyl sulfate-polyacrylamide gel electrophoresis and transferred to a nitrocellulose membrane. The E2 proteins were detected by anti-FLAG M2 antibodies.
(TIF)
