# Supplementary figures and images for: Differential Methylation of the HPV 16 Upstream Regulatory Region during Epithelial Differentiation and Neoplastic Transformation
Source: PLoS One. 2011 Sep 7;6(9):e24451. doi: 10.1371/journal.pone.0024451 (PMC3168499; doi:10.1371/journal.pone.0024451)

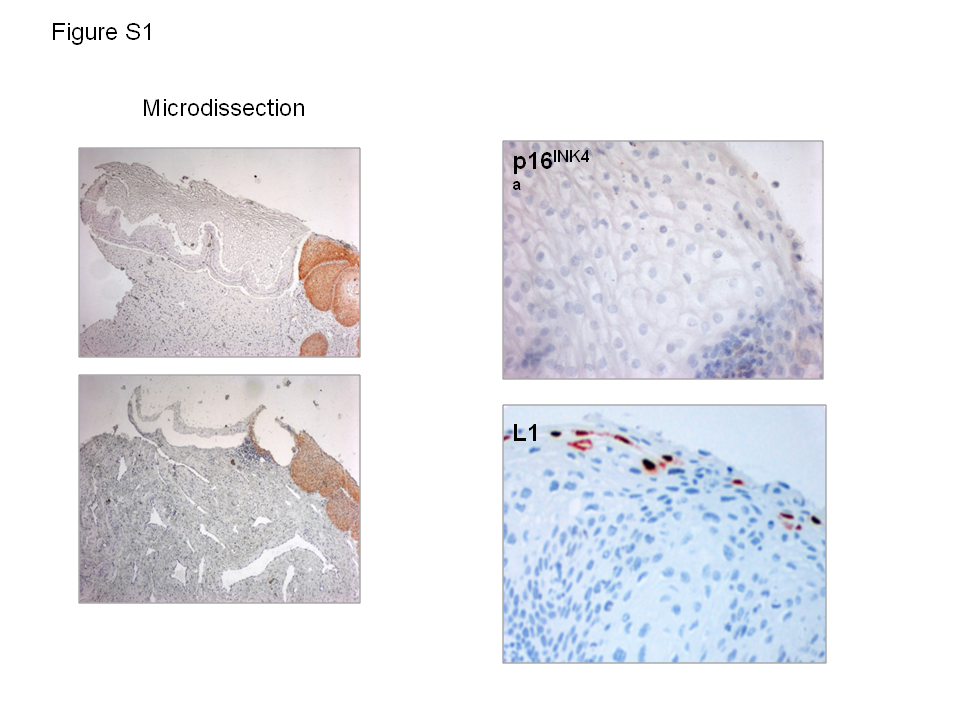

Supplement: Figure S1 — Sample N1. Regions with HPV16 permissive and transforming infections. Before and after microdissection (left upper and lower images). The p16INKa-negative region displaying koilocytes (upper,right image) and L1 expression (lower right image) as indicator for permissive infection. This area corresponds in pathology terms to a flat condyloma. (TIF) [file pone.0024451.s001.tif]

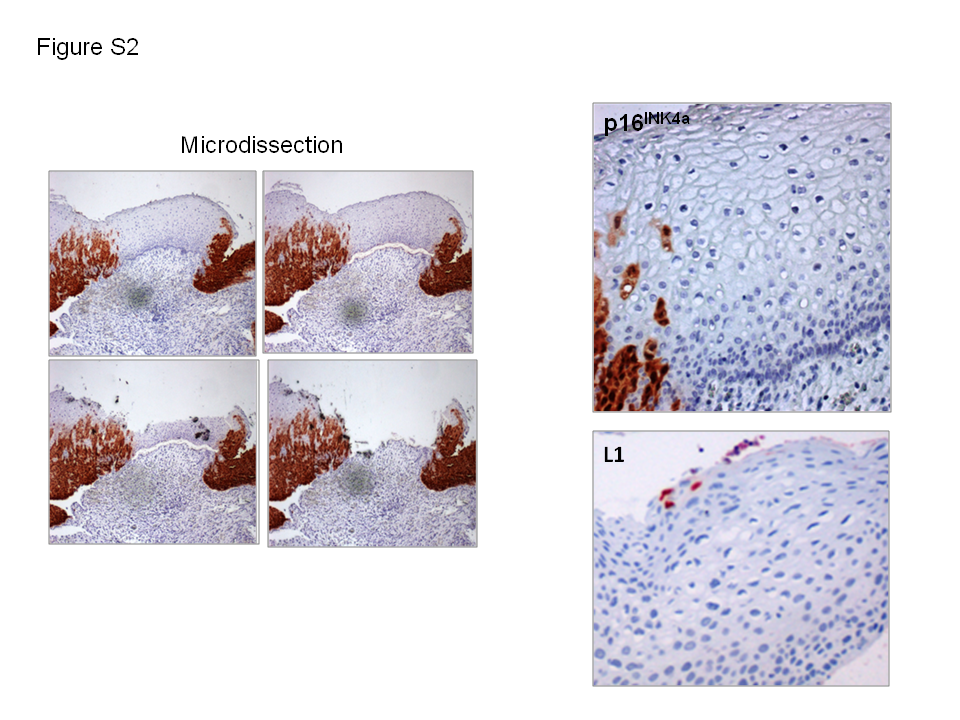

Supplement: Figure S2 — Sample N2. Region with HPV16 permissive infection. Before and after microdissection (left upper and lower images). The p16INKa -negative region displaying koilocytes (upper right image) and L1 expression (lower right image) as indicator for permissive infection. The p16INK4a-negative parts here again correspond to a flat condyloma. (TIF) [file pone.0024451.s002.tif]

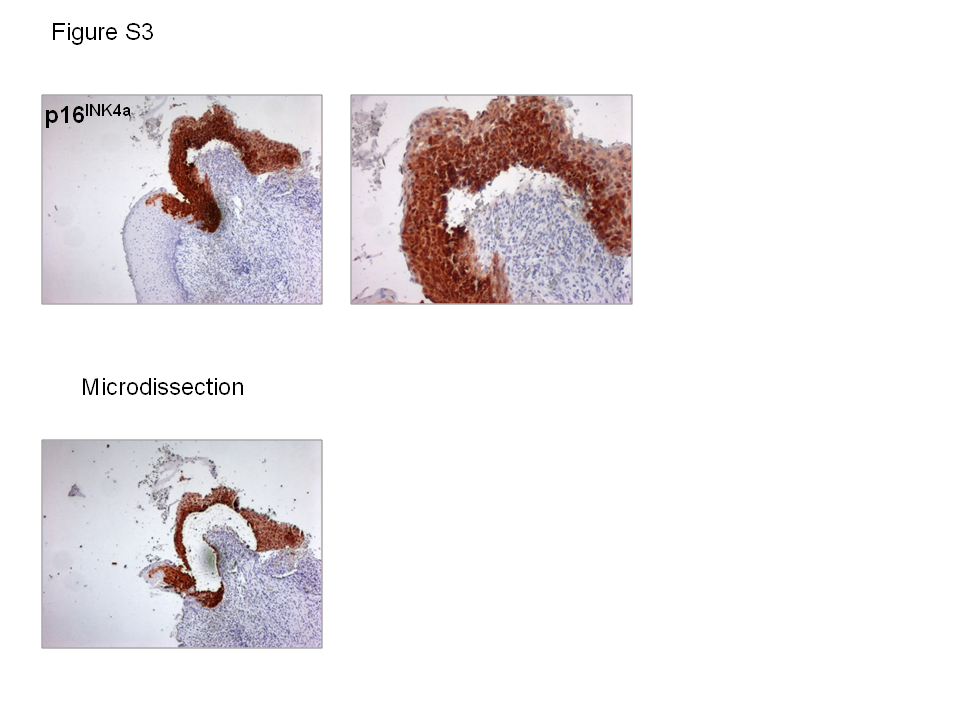

Supplement: Figure S3 — Sample N2. Region with HPV16 transforming infection. Before and after microdissection (left upper and lower images). The p16INKa positive region (right image) of transformed epithelium. (TIF) [file pone.0024451.s003.tif]

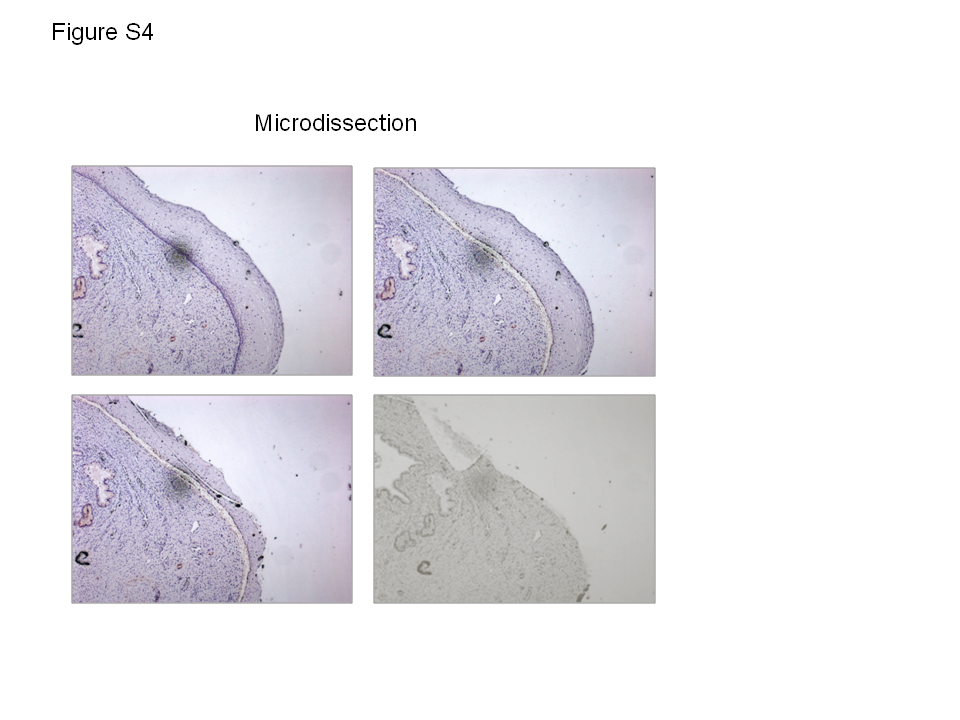

Supplement: Figure S4 — Sample N1. Region with HPV16 latent infection. Before and after microdissection. The p16INKa-negative region without morphological changes of the epithelium and no signs of the viral replication (no koilocytes and no L1 expression). (TIF) [file pone.0024451.s004.tif]

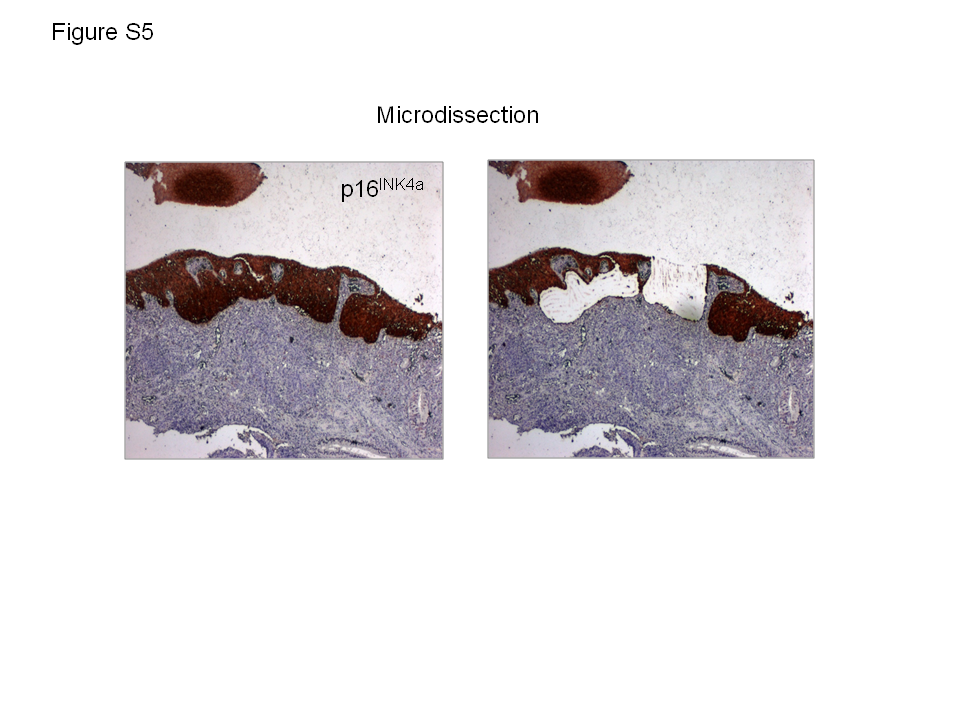

Supplement: Figure S5 — Sample N3. Region with HPV16 transforming infection. Before and after microdissection. The p16INKa-positive region (right image) of transformed epithelium. (TIF) [file pone.0024451.s005.tif]

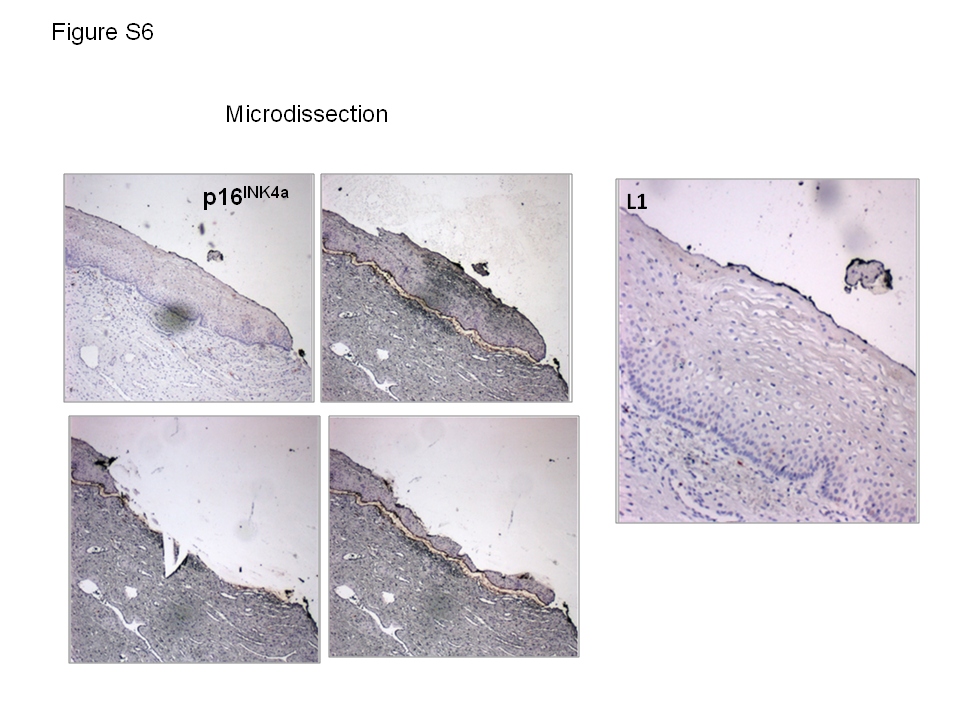

Supplement: Figure S6 — Sample N2. Region with HPV16 latent infection. Before and after microdissection. The p16INKa-negative region without morphological changes of the epithelium and no signs of the viral replication (no koilocytes and no L1 expression). (TIF) [file pone.0024451.s006.tif]

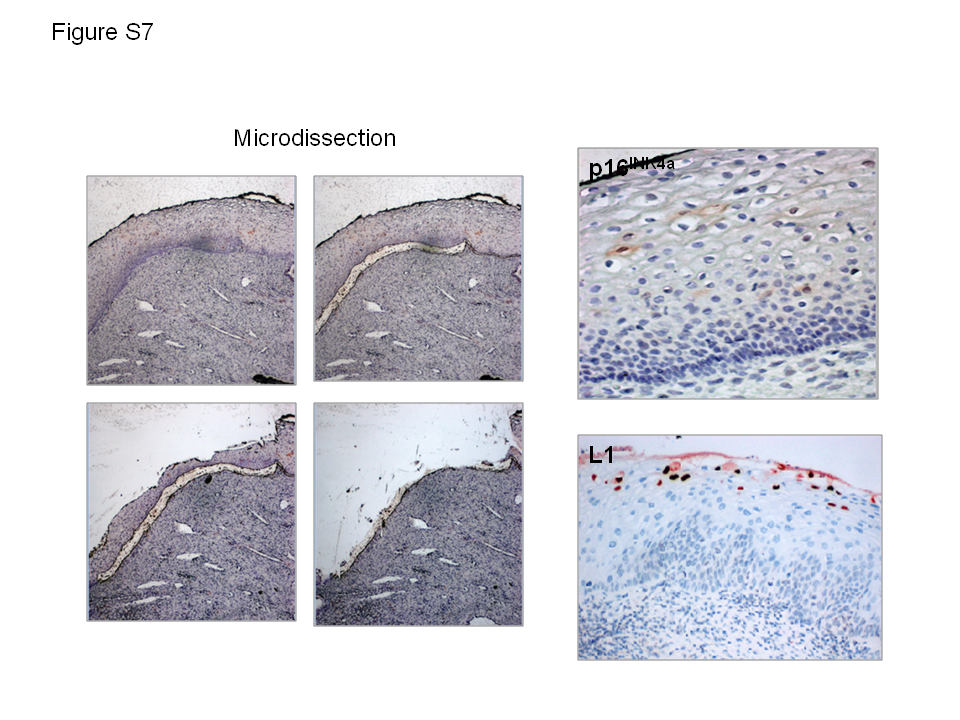

Supplement: Figure S7 — Sample N3. Region with HPV16 permissive infection. Before and after microdissection (left upper and lower images). The p16INKa-negative region displaying koilocytes (upper right image) and L1 expression (lower right image) as indicator for permissive infection, corresponding again to a flat condyloma. (TIF) [file pone.0024451.s007.tif]

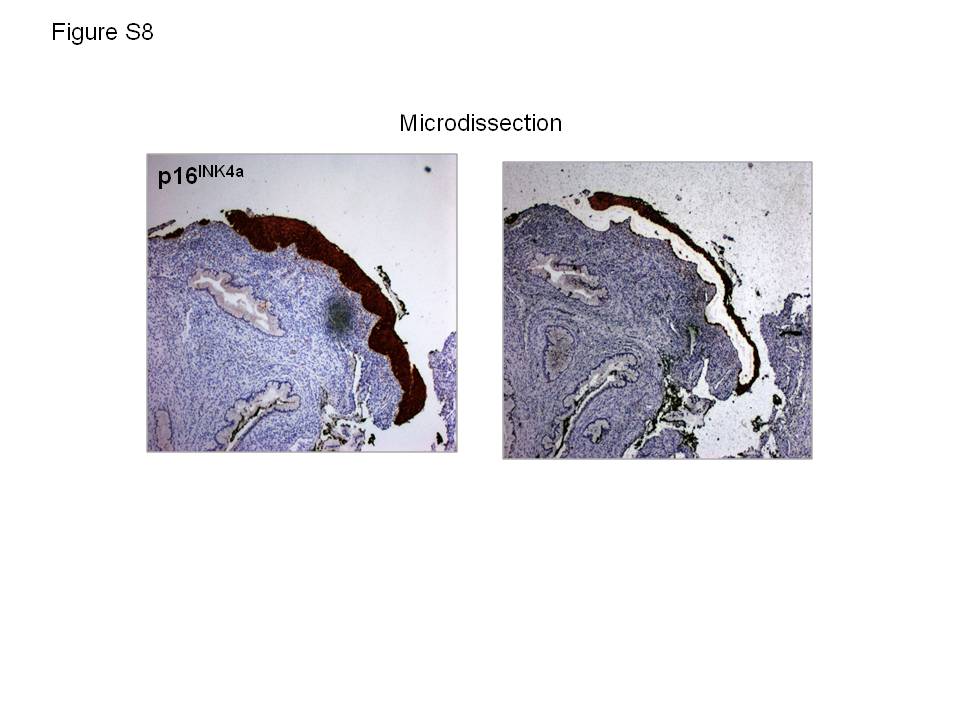

Supplement: Figure S8 — Sample N4. Region with HPV16 transforming infection. Before and after microdissection. The p16INKa -positive region (right image) of transformed epithelium. (TIF) [file pone.0024451.s008.tif]

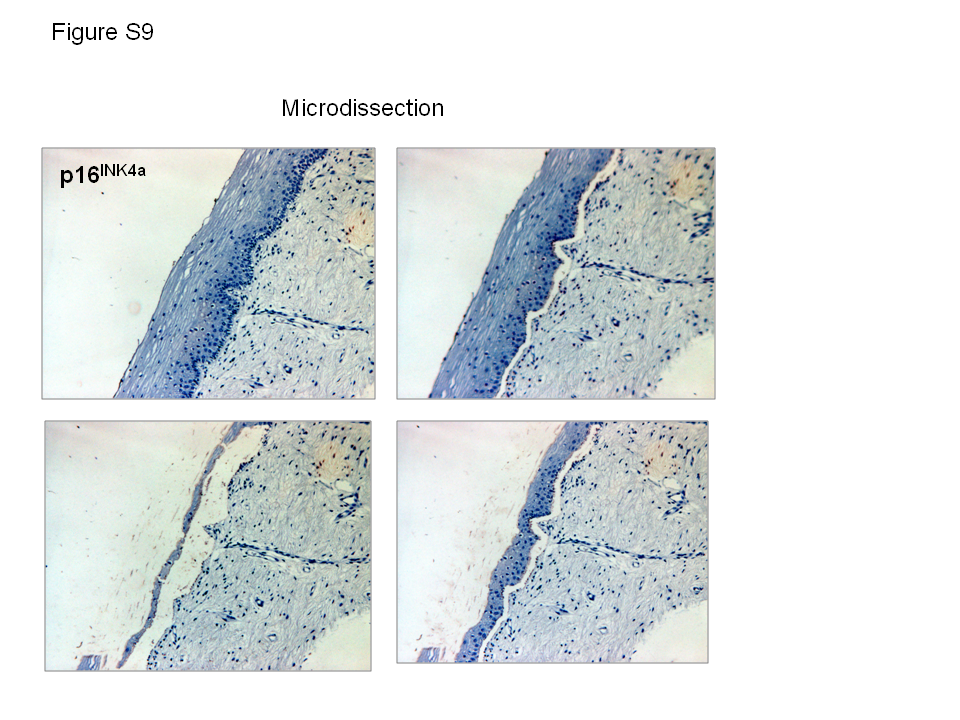

Supplement: Figure S9 — Sample N3. Region with HPV16 latent infection. Before and after microdissection. The p16INKa-negative region without morphological changes of the epithelium and no signs of the viral replication (no koilocytes and no L1 expression). (TIF) [file pone.0024451.s009.tif]

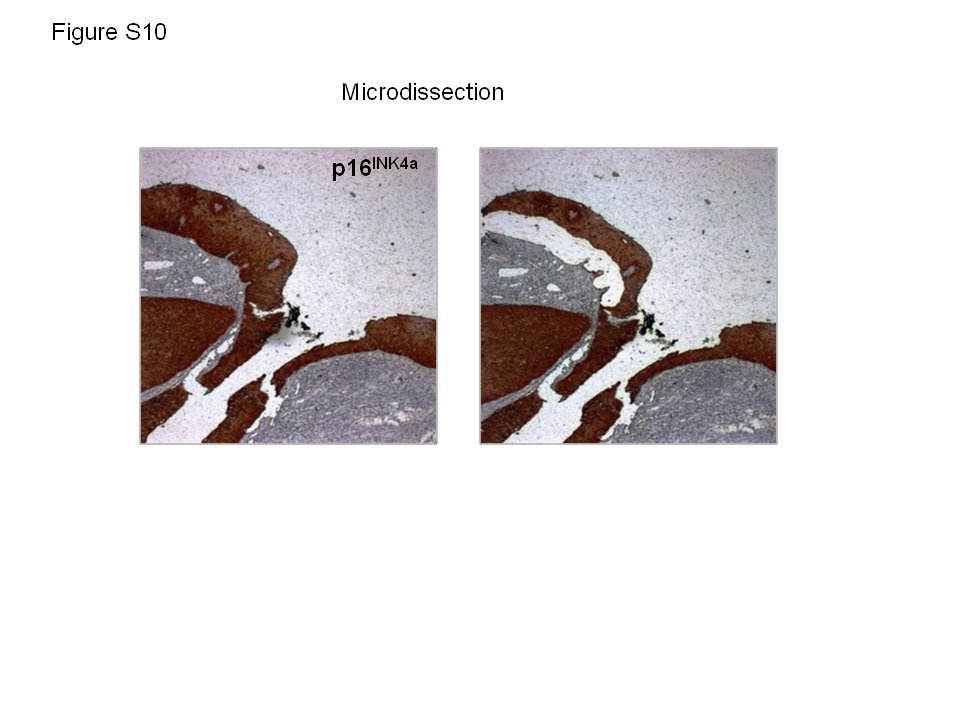

Supplement: Figure S10 — Sample N5. Region with HPV16 transforming infection. Before and after microdissection. The p16INKa-positive region (right image) of transformed epithelium. (TIF) [file pone.0024451.s010.tif]

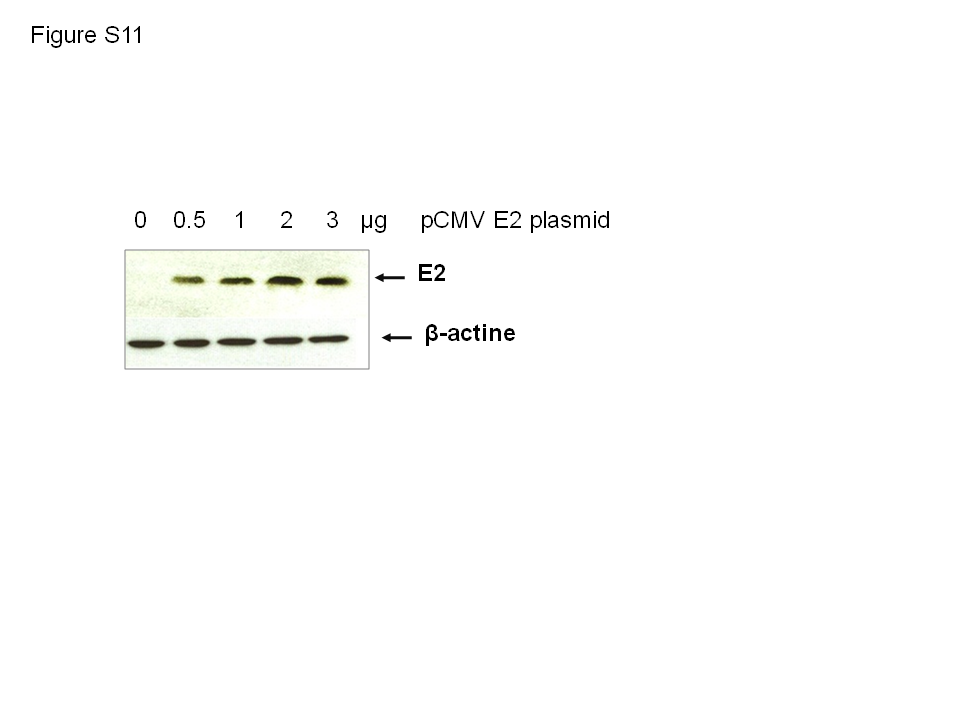

Supplement: Figure S11 — Expression of HPV-16 E2 protein in C33A cells. The HPV 16 E2 protein was expressed from the pFLAG-CMV-3 vector. After 48 h of incubation, the transfected cells were disrupted in 200 µl of lysis buffer. Forty microliters of each lysate was subjected to sodium dodecyl sulfate-polyacrylamide gel electrophoresis and transferred to a nitrocellulose membrane. The E2 proteins were detected by anti-FLAG M2 antibodies. (TIF) [file pone.0024451.s011.tif]
